# Does hypothalamic CCN3 hypersecretion confer postpartum mood disorder risk?

**DOI:** 10.1038/s41398-026-03969-9

**Published:** 2026-03-20

**Authors:** William Davies

**Affiliations:** 1 Division of Psychological Medicine and Clinical Neurosciences and Centre for Neuropsychiatric Genetics and Genomics, School of Medicine, Cardiff University, Cardiff, UK; 2 School of Psychology, Cardiff University, Cardiff, UK; 3 Neuroscience and Mental Health Innovation Institute, Cardiff University, Cardiff, UK

**Keywords:** Clinical genetics, Psychiatric disorders, Molecular neuroscience, Physiology

## Abstract

Postpartum mood disorders, of which postpartum psychosis is the most severe, occur shortly after childbirth in a proportion of mothers. These conditions can impact negatively and substantially on maternal health, mother-child bonding, and family dynamics, but their pathophysiology is poorly-understood, and treatment options are limited. Following recent research in a preclinical model, here, I propose that hypersecretion of the CCN3 protein from kisspeptin neurons of the hypothalamic arcuate nucleus in response to severe oestrogen depletion and/or abnormal calcium metabolism predisposes to adverse postpartum mental health. This novel idea reconciles many previous disparate theoretical, preclinical, and clinical findings with respect to postpartum psychopathology, and suggests that neuroendocrine and psychological processes act in combination to confer disorder risk. It also provides testable predictions: notably, that circulating CCN3 levels are positively-correlated with adverse postpartum mood symptoms in humans, and that CCN3 administration/over-expression in postpartum animal models induces relevant behavioural abnormalities. Empirical support for this hypothesis would indicate viable alternative therapeutic strategies e.g. pharmacological downregulation of CCN3.

Postpartum mood disorders (PMDs) can affect up to 40% of new mothers following childbirth [1]. Such disorders range in nature, prevalence, severity and duration but commonly encompass disabling depressive and anxiety-related symptoms which can significantly impact the affected individual, their family and loved ones, as well as impairing initial infant bonding; they are also associated with heightened risk of suicide and, in extreme cases, infanticide [1, 2]. Postpartum (or puerperal) psychosis (PP) is the rarest, and most severe, psychiatric phenotype seen in postpartum women, and is characterised by the presence of hallucinations and delusions (often infant-related), mood swings (ranging from mania to depression to extreme anxiety), disturbed sleep, and disorientation generally occurring shortly after childbirth [1]. The pathophysiology of PMDs is complex and poorly-understood, but there are likely to be common risk mechanisms operating across conditions; notably, vulnerability is thought to be partially conferred by extreme maternal age, exposure to stressors, and a biological predisposition to the large and rapid drop in circulating oestrogen, progesterone and allopregnanolone levels occurring after placental expulsion [1]. By far the biggest risk factor for PP is a previous PP episode; a prior diagnosis of bipolar disorder (or a related mood-psychotic disorder) is also associated with significantly increased PP risk [1, 2]. PMDs have been reported to be disproportionately comorbid with a number of medical conditions including thyroid conditions and pre-eclampsia (pathologically elevated blood pressure which typically occurs in late pregnancy and which can also occur postnatally) [1, 2]. PMDs have also been linked to abnormalities in the peripheral immune system, notably with respect to cytokine signalling [1, 2]. Linkage and association analyses in women with PP have implicated the chromosomal regions 16p13 and 8q24, and specific serotonergic system genes [2]. In combination with psychosocial interventions, pharmacological approaches such as the administration of antipsychotic, antidepressant, mood-stabilising or neurosteroid drugs can be efficacious in treating PMDs, but their mechanisms of action are often unclear, their efficacy is highly-variable, and there are potential side-effects and concerns with transmission of drugs to the breastfeeding child [1, 2]. Surprisingly, maternal smoking has been suggested as being protective against the development of PP [3].

Haploinsufficiency for steroid sulfatase (STS), an enzyme mediating oestrogen biosynthesis, confers postpartum depression risk [4] and potentially also PP risk [5]. Consistent with this, acute STS inhibition in postpartum mice resulted in behavioural abnormalities that could be partially alleviated by antipsychotic administration [6]. Subsequent analysis of candidate genes identified elevated brain expression of *Ccn3*/*Nov* and *Ccn2/Ctgf* (encoding the heterodimerising Cellular Communication Network factor proteins CCN3 and CCN2) as a potential mediating mechanism; *Ccn3* brain expression could be downregulated by antipsychotic administration [6]. CCN3 and CCN2 represent plausible molecular mediators of postpartum mood (discussed comprehensively in [7]): i) they are abnormally expressed in multiple animal models of postpartum mood disturbance, ii) they are highly-expressed in relevant brain regions and *Ccn3* expression increases significantly in the hypothalamus from late-pregnancy into the postpartum period in mice, iii) they are thought to mediate depressive, anxiety-related, and psychotic traits in part via myelination effects exerted through Discoidin Domain Receptor Tyrosine Kinase 1 (DDR1), and iv) the *CCN3* gene is located at 8q24. In addition to their likely behavioural effects, there is strong evidence that CCN3 and CCN2 regulate blood pressure with both being implicated in pre-eclampsia pathophysiology, and CCN3 has also been associated with sleep disturbance through effects on obstructive sleep apnoea risk [7, 8].

The hypothesis presented below regarding the biological basis of PMDs is based upon a groundbreaking finding recently published by Babey and colleagues [9]. Their work, in mouse models, elegantly showed that, in order to maintain bone integrity in the face of oestrogen depletion and calcium loss due to lactation/suckling in the postpartum period, oestrogen receptor-expressing kisspeptin neurons of the hypothalamic arcuate nucleus of the maternal brain increase expression of the CCN3 protein and secrete it into the circulation where it acts as an osteoanabolic factor. Whether this compensatory mechanism also exists in humans is an open question, but the fact that CCN3 plasma levels are significantly associated with bone mineral density and fracture frequency [10] suggests that it probably does. CCN3 upregulation during lactation has also recently been reported to occur normally in limbic regions of the mouse brain underlying aspects of maternal behaviour, notably the posterodorsal division of the medial amygdala [11, 12].

Briefly, I hypothesise that acute hypersecretion of CCN3 from kisspeptin-positive neurons of the maternal hypothalamus occurs in a proportion of postpartum women in response to extreme oestrogen depletion (e.g. as in the case of STS deficiency), as a consequence of structural-functional abnormalities of this specific neurocircuitry, and/or in response to the impaired integration or retention of calcium ions in bone (which could develop over time, and asymptomatically, prior to the postpartum period); in these women, elevated circulating CCN3 (and CCN2) levels might then elicit the mood and neurostructural abnormalities, hypertensive phenotypes, and sleep disruption associated with PMDs (Fig. 1). Abnormal expression of CCN3 in limbic brain regions implicated in psychotic-mood disorders such as the medial amygdala [13, 14] may also occur, and contribute to psychiatric phenotypes, in these postpartum women. Of course, I recognise that PMD risk, like that of most psychiatric disorders, is likely to be underpinned by multiple complex, interacting biological and environmental factors involving multiple physiological systems e.g. hormonal, stress, and inflammation-related pathways (discussed further in [15, 16]), some of which may overlap with those proposed above, and others of which may act independently. I also appreciate that, on the basis of current limited data, there is some degree of uncertainty over whether changes in CCN protein levels in humans have causal effects on mood, myelination and sleep phenotypes, and recognise the need for further work in this area.

**Fig. 1:**
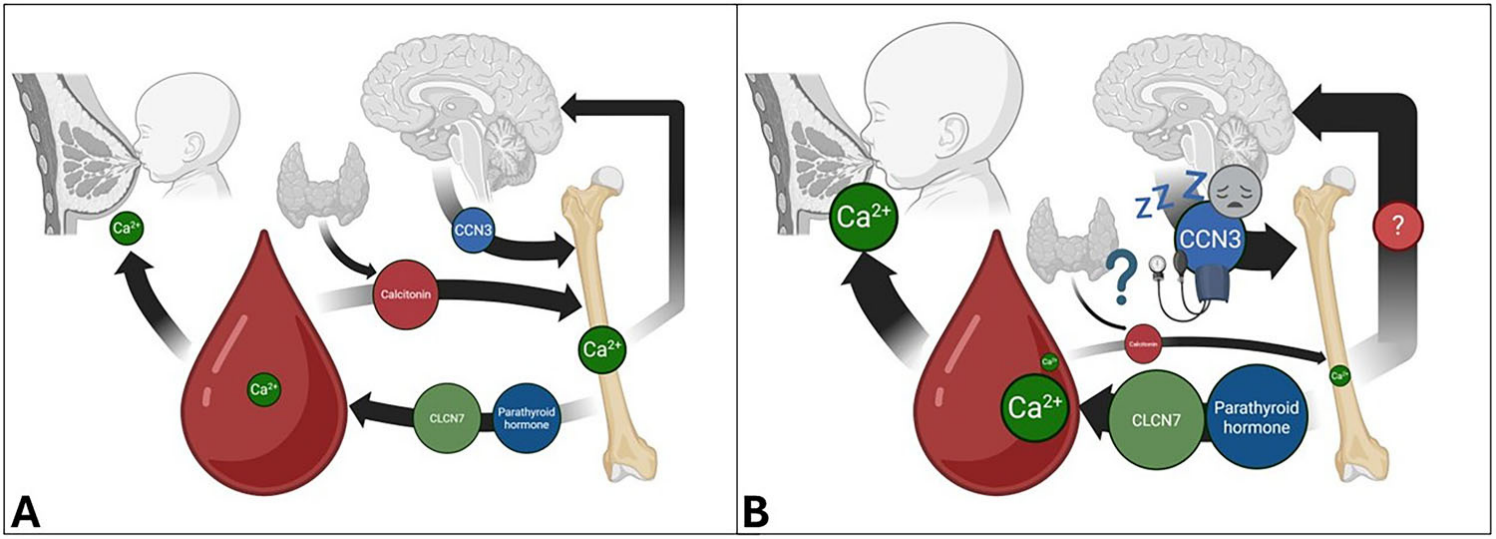
Models of the postpartum brain-bone axis in healthy women and in women at risk of mood disorder. **A** In healthy postpartum women, CCN3 is moderately-expressed from kisspeptin-positive cells of the hypothalamic arcuate nucleus and secreted into the blood where it acts as an osteoanabolic factor to mitigate calcium ion loss from the blood due to lactation/suckling and acute loss of osteoprotective oestrogen. Blood-bone calcium balance is maintained, in part, by the thyroid hormone calcitonin (indirectly promoting calcium integration from serum into bone through inhibition of bone resorption), and by parathyroid hormone and chloride voltage-gated channel 7 (CLCN7) (directly promoting bone resorption). **B** I propose that in women vulnerable to postpartum mood disorders, there is a failure to integrate and/or retain calcium ions in bone resulting from a combination of hypocalcemia, reduced calcitonin activity (as a consequence of thyroid disease), elevated CLCN7 expression, increased parathyroid hormone activity and elevated milk demand from suckling. I speculate that this culminates in compensatory hypothalamic hypersecretion of CCN3 and deleterious effects on mood, white matter microstructure/myelination, blood pressure and sleep. As noted in the main text, causal relationships between CCN protein levels and mood, myelination, and sleep phenotypes have yet to be reliably established in humans, and even if they exist, they are unlikely to be deterministic. Created in BioRender.com (https://BioRender.com/y18e045 and https://BioRender.com/i7y5fre).

Impaired integration of calcium ions into bone in the postpartum period could theoretically be due to two mechanisms: i) low availability of these ions in serum/plasma (‘hypocalcemia’) as a consequence of dietary deficiency, low gastrointestinal absorption, underlying medical conditions, or excessive demand from lactation/suckling and/or ii) deficiencies in the biochemical systems which directly or indirectly facilitate the transfer of calcium ions from serum to bone, notably in the levels or function of the thyroid hormone calcitonin (an inhibitor of bone resorption). In contrast, impaired retention of calcium ions in bone (i.e. excessive resorption), and consequent hypercalcemia, could be due to hyperactivity of biochemical systems mediating the transfer of calcium ions from bone to serum; these might include elevated levels (or function) of parathyroid hormone (PTH) and Vitamin D, or over-expression of the chloride voltage-gated channel 7 [17], the gene for which (*CLCN7*) is located at 16p13.3. Bone resorption is influenced by age [18], serotonergic function [19, 20], and inflammatory processes [21].

There is existing evidence in support of these ideas. New neuroimaging evidence has shown altered inferior tubular subunit volume (a region subsuming the arcuate nucleus), and sensitivity of this measure to antipsychotic medication, in patients with mood-psychosis spectrum conditions [22], while data from adult female mice has shown that the activity of kisspeptin-positive arcuate nucleus neurons is impacted by psychosocial stress [23] and is linked to circadian rhythm behaviours [24]. Postpartum hypocalcemia in cattle (arising from acute high milk demand by calves) is associated with ‘milk fever’, a behavioural analogue of postpartum psychosis characterised by hypersensitivity, excitability/restlessness, and abnormal motor function [25], acute hypocalcemia in women of reproductive age can be associated with psychosis [26] and dietary calcium deficiency has been linked to increased postpartum depression risk [27]. In pigs, offspring-directed maternal aggression, a proposed correlate of PP [28], tends to be greater with larger litters i.e. where there is a greater milk demand on the mother [29, 30]. Similarly, in humans, twin births are more strongly associated with severe maternal mental illness symptoms than singleton births [31]. Lithium salts, commonly administered to control postpartum mood swings, can elevate plasma calcium ion levels [32]. In patients with bipolar disorder, calcitonin supplementation has been proposed to be efficacious in treating acute manic symptoms [33]. Hypercalcemia has been described in a group of patients exhibiting PP [34] and primary hyperparathyroidism has been linked to bipolar and psychotic disorders in rare cases, with psychiatric manifestations being reduced through parathyroidectomy [35]. Moreover, PTH levels are higher in individuals diagnosed with bipolar disorder than in healthy controls [36] and positively correlate with symptom severity [37]. Smoking tends to boost calcitonin levels and diminish PTH levels [38], and these actions may explain its apparent protective effect against PP. Finally, there is an increasingly-recognised link between psychotic and mood disorders (notably schizophrenia and bipolar disorder) and bone metabolism, even in drug-naïve patients [39, 40].

This hypothesis generates several clear and testable predictions that might be addressed in future work. First, that, compared to matched healthy postpartum individuals, individuals experiencing PMDs (or animals displaying abnormal maternal behaviours) will display: elevated levels of circulating CCN3 and CCN2 proteins, abnormal serum calcium and Vitamin D levels, thyroid pathology resulting in reduced levels/activity of circulating calcitonin, elevated levels/activity of parathyroid hormone, increased peripheral markers of inflammation, and a heightened exposure to milk demand from their offspring. Additionally, I would expect the latter groups to exhibit structural and functional differences from the former group with respect to the arcuate nucleus and the kisspeptin-positive neurons contained therein. The aforementioned measures would be expected to be correlated with one another, and also with postpartum symptom severity (mood, blood pressure, and degree of sleep disturbance). Second, in future genetic analyses, I would anticipate enrichment of PMD risk variants in candidate genes (*CCN3*, *CCN2*, *DDR1*, *CALCA*/*CALCR*, *PTH*/*PTH1R* and *CLCN7*) or in relevant metabolic pathways (e.g. ‘calcium ion homeostasis’ or ‘parathyroid hormone synthesis, secretion and action’). Although the latest genomewide association studies (GWAS) of PMDs have yielded limited significant findings [41–43], it is interesting to note that *HMCN1*, a gene previously implicated in postpartum mood symptoms [44], encodes a protein important in matrix production by osteoblasts in demineralised bone [45]. Moreover, whole genome sequencing of cases has recently implicated loss-of-function variants in the genes *HMGCR* (encoding 3-Hydroxy-3-Methylglutaryl-CoA Reductase) and *DNMT1* (encoding DNA Methyltransferase 1) in PP risk [46]. Interestingly, statins, which act as HMGCR inhibitors, promote *CCN3* expression in endothelial cells [47], modulate the activity of CCN3-expressing regulatory T-cells previously implicated in PP risk via effects on myelination [48–50], and influence bone metabolism [51]. DNMT1 has also been characterised as a regulator of bone metabolism [52].

In order to indicate causality, I would expect the following manipulations in postpartum subjects to result in behavioural and physiological abnormalities of relevance to PMDs: administration of CCN3 or PTH (or modulation of activity of kisspeptin-positive neurons resulting in CCN3 hypersecretion), and calcitonin depletion/inhibition. If these predictions are supported experimentally, alternative interventions (e.g. promoting bottle- rather than breastfeeding for some women) or pharmacological treatment approaches (e.g. downregulation of CCN3 or calcitonin supplementation) might be trialled. Finally, CCN3 axis dysfunction may feasibly also contribute towards the observed increased rate of psychiatric diagnoses in the perimenopausal period in women [53]; like the postpartum period, the perimenopausal period is characterised by oestrogen depletion and changes in bone metabolism [54], and adverse mood symptoms and bone health measures appear to correlate within it [55].
